# Auditory outcomes and predictors following ossiculoplasty in cholesteatoma surgery: a retrospective analysis

**DOI:** 10.3389/fsurg.2025.1630444

**Published:** 2025-11-14

**Authors:** Alexandre Karkas, Fabien Tinquaut, Asimakis Asimakopoulos, Brandon Grondier, Pierre Bertholon, Ines Abounaidane

**Affiliations:** 1 Department of Otolaryngology-Head & Neck Surgery, University Medical Center of Saint-Etienne, Saint-Etienne, France; 2 School of Medicine, University Jean Monnet, Saint-Etienne, France; 3 Department of Public Health Service, University Medical Center of Saint-Etienne, Saint-Etienne, France

**Keywords:** cholesteatoma surgery, ossiculoplasty, auditory results, predictors, hearing outcome

## Abstract

**Introduction:**

Middle ear cholesteatoma causes bone/ossicular erosion. This study aimed to analyze auditory outcomes after ossiculoplasty in cholesteatoma surgery and to identify predictors of hearing outcomes related to middle ear or surgery.

**Methods:**

A retrospective study was conducted on patients who underwent ossiculoplasty during cholesteatoma surgery (2019–2024). Preoperative, short-term (2-month), and midterm (8-month) postoperative audiograms were analyzed. Potential pre-/postoperative parameters influencing hearing were sought.

**Results:**

Eighty-eight cases were included (20 pediatric, 68 adult). There were 56 primary surgeries, and 28 patients had preoperative cholesteatoma complications. In case of present stapes, a stapes–cartilage augmentation was mostly performed, followed by a partial ossicular replacement prosthesis. In case of absent stapes, a total ossicular replacement prosthesis was used. There were 10 postoperative complications (1 prosthesis extrusion) and 16 residual cholesteatomas (1–3 years). Short-term mean postoperative gain in bone conduction (ΔBC) was 0.3 dB, while midterm ΔBC was 1.4 dB. Short-term mean postoperative gain in air conduction (ΔAC) was 3.4 dB, while midterm ΔAC was 4.5 dB. Short-term mean postoperative gain in air–bone gap (ΔABG) was 3.4 dB, while midterm ΔABG was 3.7 dB. Preoperatively, younger age favorably influenced AC and BC, and the presence of stapes favorably influenced ABG. Postoperatively, regarding midterm ΔABG, the absence of posterior tympanotomy was a predictor of good hearing outcome. Considering the midterm postoperative ABG alone, the absence of mastoidectomy was a favorable predictive factor. Regarding midterm ΔAC, primary surgery was a predictor of a good hearing outcome. Considering postoperative AC alone, there were three favorable predictive factors, namely, younger age, primary surgery, and absence of mastoidectomy. There was no predictive factor for midterm ΔBC. The malleus handle had no effect on auditory results.

**Discussion:**

Postoperative auditory results of our study are fair, given the preoperative aggressiveness/extension of cholesteatoma, but were comparable to a few other studies, as were the rates of postoperative complications and residual disease. Younger age and presence of stapes were predictive of better preoperative hearing. Postoperatively, younger age, absence of mastoidectomy, absence of posterior tympanotomy, and primary surgery were predictors of good hearing outcome. Results in the literature are highly variable, sometimes contradictory. This stems from the diversity of disease extension, surgical techniques, and materials used in ossiculoplasty.

## Introduction

Middle ear cholesteatoma is a mass formed by keratinizing squamous epithelium in the tympanic cavity and/or mastoid and subepithelial connective tissue and by progressive accumulation of keratin debris. Recurrent infections and inflammatory reactions within the subepithelial connective tissue by cholesteatoma contribute to bone resorption in the adjacent areas (1). Middle ear ossicles can thus be eroded, particularly the incus, then the stapes (2). Treatment of cholesteatoma is surgical. The primary goal of surgery is complete resection of cholesteatoma and avoidance of residual disease. The secondary goals are reconstruction (tympanic membrane, canal wall, atticotomy) and hearing restoration; it is now well established that ossiculoplasty can be performed as part of a single-stage operation (3, 4). In this regard, there are some knowledge gaps and discrepancies in the literature concerning hearing outcomes and their predictive factors in cholesteatoma surgery. This study aimed to analyze auditory results following cholesteatoma surgery with ossiculoplasty and identify potential prognostic factors related to middle ear status or surgical technique.

## Materials and methods

This is a retrospective study conducted on patients undergoing ossiculoplasty performed as part of cholesteatoma surgery between 2019 and 2024 (6 years) at our university medical center. The study population involved pediatric and adult patients requiring surgery for cholesteatoma associated with a non-continuous ossicular chain. Patients having cholesteatoma with a continuous ossicular chain and not undergoing ossiculoplasty were not included. Data were collected from paper medical records and computerized data, enabling the collection of the required clinical and audiometric information. Preoperative, as well as early (2 months) and midterm (6–9 months, median 8 months), postoperative audiograms were selected for analysis. We collected the puretone average (PTA) of every patient by calculating the mean air conduction (AC) and mean bone conduction (BC) at 500, 1,000, 2,000, and 4,000 Hz, thus adhering to the guidelines of the Committee on Hearing and Equilibrium for the evaluation of results of treatment of conductive hearing loss (5). We then evaluated the conductive hearing loss by calculating the mean air–bone gap: ABG = mean AC − mean BC. We defined the short-term and midterm auditory change (Δ) as the mean preoperative thresholds minus the mean short-term and midterm postoperative thresholds, respectively (ΔBC, ΔAC, and ΔABG). Patients lost to follow-up (not having *at least* one audiometry at 2 months or at 8 months) were excluded from the analysis. All patients were operated on in the same institution by the same experienced surgeon. The choice of ossiculoplasty depended on anatomical criteria, namely, the presence or absence of the stapes superstructure. We analyzed the functional results of ossiculoplasty in cholesteatoma and tried to identify pre- and postoperative parameters associated with audiometric data: age, surgical technique, presence or absence of the malleus handle and stapes superstructure, type of ossiculoplasty, occurrence of complications, mastoid obliteration, status of the middle ear mucosa, and primary or iterative nature of the procedure.

All variables were described using means (standard deviation, SD) and medians (quartiles Q1–Q3) for quantitative variables and sample size (*N*, %) for qualitative variables. The paired Student’s *t*-test was used to analyze the gain in air–bone gap depending on the frequency and for the comparison of short-term and midterm auditory results. The Kruskal–Wallis test was used to evaluate the significance of predictive factors of hearing outcome (non-normality of quantitative variables). The Student’s *t*-test was also used to assess whether midterm hearing results (BC, AC, and ABG) differed *significantly* from preoperative results. The risk *α* was set at 5%. The R software (version 4.2.1) was used for statistical analyses.

## Results

### General data

Eighty-eight cases were eligible for inclusion and data analysis. Patient age ranged from 5.2 to 85 years (mean 36.8 years, SD 21.5). Twenty patients were younger than 18 years (22.7%), and 68 were adults (77.3%). Among the adults, 49 were aged ≤60 years (55.7%) and 19 were aged >60 years (21.6%). A total of 43 right ears and 45 left ears were operated on in 83 patients. Fifty-six ears were operated on for the first time and 32 ears for ≥2nd time, mostly performed elsewhere. During the period of the study, two patients were operated on twice in our institution, one patient operated thrice, and another was operated on both ears sequentially. Patients with congenital cholesteatoma were not included in this study, as its pathophysiology is different from acquired cholesteatoma. In 18 cases, no mastoidectomy was performed, whereas in the remaining 70 cases, a canal-wall-up (CWU) mastoidectomy was added to the tympanoplasty. Patients undergoing canal-wall-down (CWD) mastoidectomy were not included due to the very small number of cases (only three cases in 6 years). In the CWU group, a posterior tympanotomy was added in 28 of the 70 cases. All patients benefited from an ossiculoplasty at the end of surgery, after resection of the cholesteatoma and reconstruction of the tympanic membrane and atticotomy. In patients who underwent posterior tympanotomy, ossiculoplasty was carried out either through this approach after repositioning the tympanomeatal flap or through the ear canal, with the stability of the ossiculoplasty confirmed through posterior tympanotomy. In the case of a present stapes superstructure (60 patients), when the distance between the stapes head and the cartilage tympanic graft was very small, a direct assembly (stapes augmentation) was preferred, often by adding a second thin cartilage plate onto the stapes head (32 patients). If the height was significant, a partial ossicular replacement prosthesis (PORP) was mainly used (21 patients). Incus transposition was less often used and was selected in some cases where the incus was still usable and not invaded by keratin (4 patients). Incudo-stapedial joint bridging with bone cement (OtoMimix®, Olympus America Inc.) was seldom used, in case of erosion of only the lenticular process of the incus (three patients). If the stapes superstructure was absent (28 patients), a total ossicular replacement prosthesis (TORP) was used in all cases. The ossicular prostheses (PORP and TORP) were all made of titanium and had a shaft of 0.2 mm diameter. They were mainly supplied by Kurz® (Heinz Kurz GmbH, Dusslingen, Germany) and more recently by MED-EL® (MED−EL Elektromedizinische Geräte GmbH, Innsbruck, Austria); the prostheses of both manufacturers have substantially similar designs and properties (Table 1).

**Table 1:** General data.

Cases, *N* = 88	Modality	*N* (%)
Age (years)	Mean (SD)	36.8 years (21.5)
	Min–max	5.2–85 years
Age by subgroups	[0–18 years]	20 (22.7%)
	[18–60 years]	49 (55.7%)
	[60–100 years]	19 (21.6%)
Operated ear	Right	43 (48.9%)
	Left	45 (51.1%)
Surgery	Primary	56 (63.6%)
	Revision	32 (36.4%)
Mastoidectomy	Not performed	18 (20.5%)
	CWU	70 (79.5%)
Posterior tympanotomy in CWU mastoidectomy	Not performed	42 (60%)
	Performed	28 (40%)
Type of ossiculoplasty	TORP	28 (31.8%)
	Stapes–cartilage augmentation	32 (36.4%)
	PORP	21 (23.9%)
	Incus transposition	4 (4.5%)
	OtoMimix bridging	3 (3.4%)
Middle ear mucosa	Normal	69 (78.4%)
	Inflammatory	19 (21.6%)
Malleus at the end of surgery	Present	76 (86.4%)
	Absent	12 (13.6%)
Stapes superstructure	Present	60 (68.2%)
	Absent	28 (31.8%)
Preoperative complications	Facial canal erosion	11
	Lateral semicircular canal fistula	8
	Temporal lobe encephalocele	4
	Subperiosteal abscess	3
	Sigmoid sinus thrombophlebitis	2
	Total	28 (31.8%)
Postoperative complications	Facial paralysis Grade III (H–B)	1
	Worsening of preexisting FP	1
	Medial stenosis of the EAC	3
	Transient vertigo	2
	Formation of cholesterol granuloma	1
	Superinfection with otorrhea	1
	Extrusion of titanium prosthesis	1
	Total	10 (11.4%)
Mastoid obliteration after CWU mastoidectomy	Yes	54 (77.1%)
	No	16 (22.9%)
Residual cholesteatoma	Yes	16 (18.2%)
		Pediatrics 6/20 (30%)
		Adults 10/68 (14.7%)
	No	72 (81.8%)

CWU, canal-wall-up; TORP, total ossicular replacement prosthesis; PORP, partial ossicular replacement prosthesis; H–B, House–Brackmann scale of facial paralysis; FP, facial paralysis; EAC, external acoustic canal.

The mucosa of the middle ear was inflammatory in 19 patients, and the malleus handle was absent at the end of surgery in 12. Tympanic grafting was always performed with cartilage and perichondrium, chiefly from the concha (69 patients) and less often from the tragus (19 patients), when the surgical approach was endomeatal or endaural. Preoperatively, 25 patients presented with one or more complications of the cholesteatoma; 3 patients had two complications, hence 28 complications in total, among which 22 were Stage III (extracranial) and 6 were Stage IV (intracranial) complications according to the European Academy of Otology and Neuro-Otology / Japanese Otological Society (EAONO/JOS) staging (1). Mastoid cavity obliteration with GlassBone® Injectable Putty (Noraker, Lyon, France) was performed in 54 of the 70 cases of CWU mastoidectomy. Postoperatively, 10 patients had complications related to surgery. We observed a case of extrusion of the TORP at 18 months (1.1%). Apart from the prosthesis extrusion, there were six major complications among the remaining nine cases. One patient developed facial paralysis (FP) (House–Brackmann Grade III) due to a dehiscent facial nerve invaded by cholesteatoma; this FP improved to Grade II within 3 months. Another preexisting FP worsened postoperatively but recovered to its initial level 4 months after surgery. There were also 1 case of cholesterol granuloma formation, 3 cases of medial stenosis of the external acoustic canal, and 16 cases of residual cholesteatoma which were diagnosed on MRI between 1 and 3 years postoperatively and necessitated reintervention. Among these, six patients were aged below 18 years (6/20 pediatric cases = 30% rate of recidivism) and 10 were adults (10/68 adult cases = 14.7%).

### Auditory results

Regarding the early or short-term postoperative auditory results (at 2 months), 86 charts out of 88 could be analyzed, as two patients did not have their audiometry at this time period. As for the midterm postoperative auditory results (at 8 months), 76 charts out of 88 could be analyzed, because 12 patients had their audiometries much later (Table 2).

**Table 2:** Auditory results: preoperative, short-term postoperative, and midterm postoperative.

Parameter mean (SD)	Preoperative (dB)	Short-term postoperative (dB)	Short-term delta (dB)	Midterm postoperative (dB)	Midterm delta (dB)	*p*-value
BC	18.6 (11.7)	18.3 (12.4)	0.3 (8.8)	16.2 (10.9)	1.4 (9.3)	0.24
AC	49.7 (17.2)	46.6 (17)	3.4 (13.6)	44.6 (16.9)	4.5 (15.2)	0.089
ABG	29.7 (13.3)	26.7 (11.4)	3.4 (13.2)	26.2 (10.7)	3.7 (13.9)	**0**.**04**

BC, bone conduction (mean and standard deviation SD); AC, air conduction; ABG, air–bone gap; delta (Δ), preoperative thresholds minus postoperative thresholds. For example, short-term ΔBC = preoperative BC − short-term postoperative BC. Midterm postoperative improvement (Δ) was significant for ABG, but not for BC or AC (Student’s *t*-test).

Bold values are statistically significant (*p* < 0.05).

**Figure 1:**
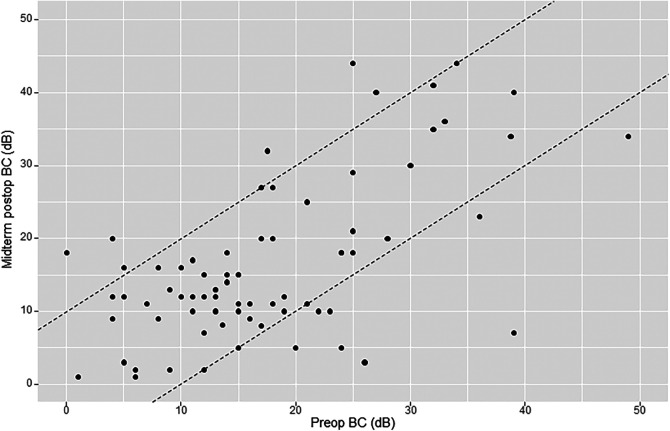
Amsterdam hearing evaluation plot (AHEP) comparing preoperative bone conduction (BC) thresholds with midterm postoperative BC thresholds. The two diagonal lines enclose cases where BC changed <10 dB. Points onto and above the lines (to the left) show an increase in BC ≥10 dB postoperatively (sensorineural hearing loss), whereas points onto and below the lines (to the right) show a decrease in BC postoperatively ≥10 dB (sensorineural hearing gain). An imaginary line crossing amidst the two diagonal lines (line zero) reflects no change in BC.

The mean preoperative bone conduction (BC) level was 18.6 dB (SD 11.7). Early mean postoperative BC was 18.3 dB (SD 12.4). Early postoperative BC remained practically unchanged because the gain (early or short-term ΔBC) was 0.3 dB (SD 8.8). Midterm mean postoperative BC was 16.2 dB (SD 10.9). Midterm postoperative gain (midterm ΔBC) was 1.4 dB (SD 9.3). Of the 76 patients analyzed for midterm postoperative BC, there were 8 cases (10.5%) of worsening ≥10 dB, 12 cases (15.8%) of improvement ≥10 dB, and 56 cases (73.7%) of BC change within 10 dB (Figure 1). We defined sensorineural hearing loss (SNHL) as an increase in BC >10 dB: five patients (5.7%) had SNHL at 8 months postoperatively. There was no case of dead ear in this study.

**Figure 2:**
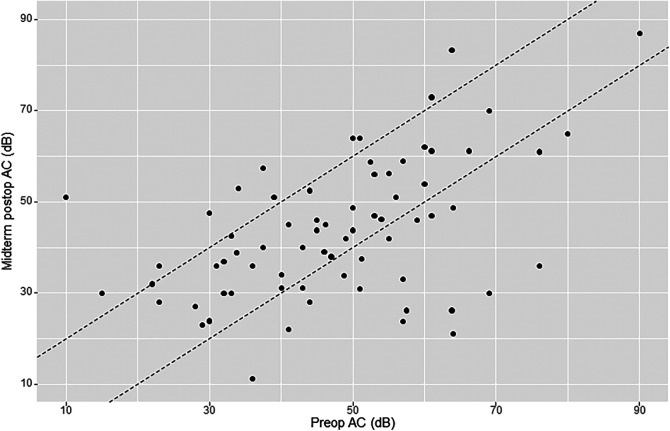
AHEP comparing preoperative air conduction (AC) thresholds with midterm postoperative AC thresholds. The two diagonal lines enclose cases where AC changed <10 dB. Points onto and above the lines (to the left) show an increase in AC ≥10 dB postoperatively (hearing loss), whereas points onto and below the lines (to the right) show a decrease in AC postoperatively ≥10 dB (hearing gain). An imaginary line crossing amidst the two diagonal lines (line zero) reflects no change in AC.

The mean preoperative air conduction (AC) level was 49.7 dB (SD 17.2). Early mean postoperative BC was 46.6 dB (SD 17). Early postoperative gain (early or short-term ΔAC) was 3.4 dB (SD 13.6). Midterm mean postoperative AC was 44.6 dB (SD 16.9). Midterm postoperative gain (midterm ΔAC) was 4.5 dB (SD 15.2). Of the 76 patients analyzed for midterm postoperative AC, there were 13 cases (17.1%) of worsening ≥10 dB, 20 cases (26.3%) of improvement ≥10 dB, and 43 cases (56.6%) of AC change within 10 dB (Figure 2).

**Figure 3:**
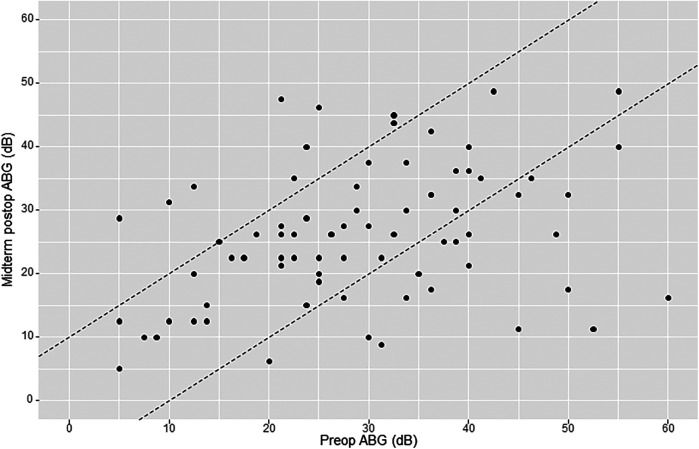
AHEP comparing preoperative air–bone gap (ABG) levels with midterm postoperative ABG levels. The two diagonal lines enclose cases where ABG changed <10 dB. Points onto and above the lines (to the left) show an increase in ABG ≥10 dB postoperatively (hearing loss), whereas points onto and below the lines (to the right) show a decrease in ABG postoperatively ≥10 dB (hearing gain). An imaginary line crossing amidst the two diagonal lines (line zero) reflects no change in ABG.

Mean preoperative air–bone gap (ABG) was 29.7 dB (SD 13.3). Early mean postoperative ABG was 26.7 dB (SD 11.4). Early postoperative gain (early or short-term ΔABG) was 3.4 dB (SD 13.2). Midterm mean postoperative ABG was 26.2 dB (SD 10.7). Midterm postoperative gain (midterm ΔABG) was 3.7 dB (SD 13.9). Of the 76 patients analyzed for midterm postoperative ABG, there were 10 cases (13.2%) of worsening ≥10 dB, 20 cases (26.3%) of improvement ≥10 dB, and 46 cases (60.5%) of ABG change within 10 dB (Figure 3).

Using the Student’s *t*-test, the midterm postoperative improvement (Δ) was significant for ABG (*p* = 0.04), but not for BC (0.24) or for AC (0.089).

**Figure 4:**
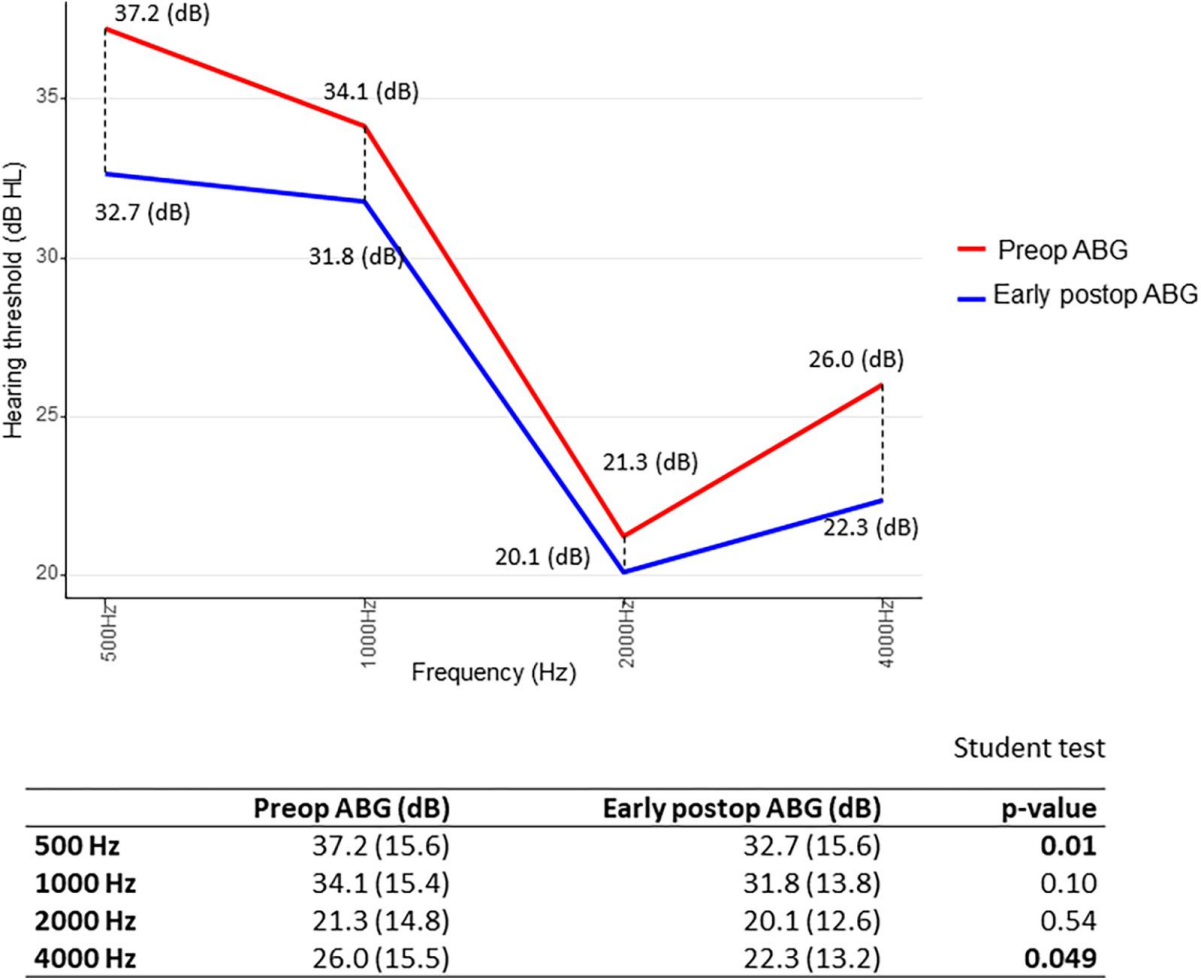
Short-term gain in air–bone gap (ΔABG) depending on frequency distribution. The *p*-value was calculated by comparing preoperative (red) and early postoperative (blue) air–bone gap (ABG) values at each frequency for each patient. We then determined whether the mean difference significantly differed from zero. Paired Student’s *t*-test.

**Figure 5:**
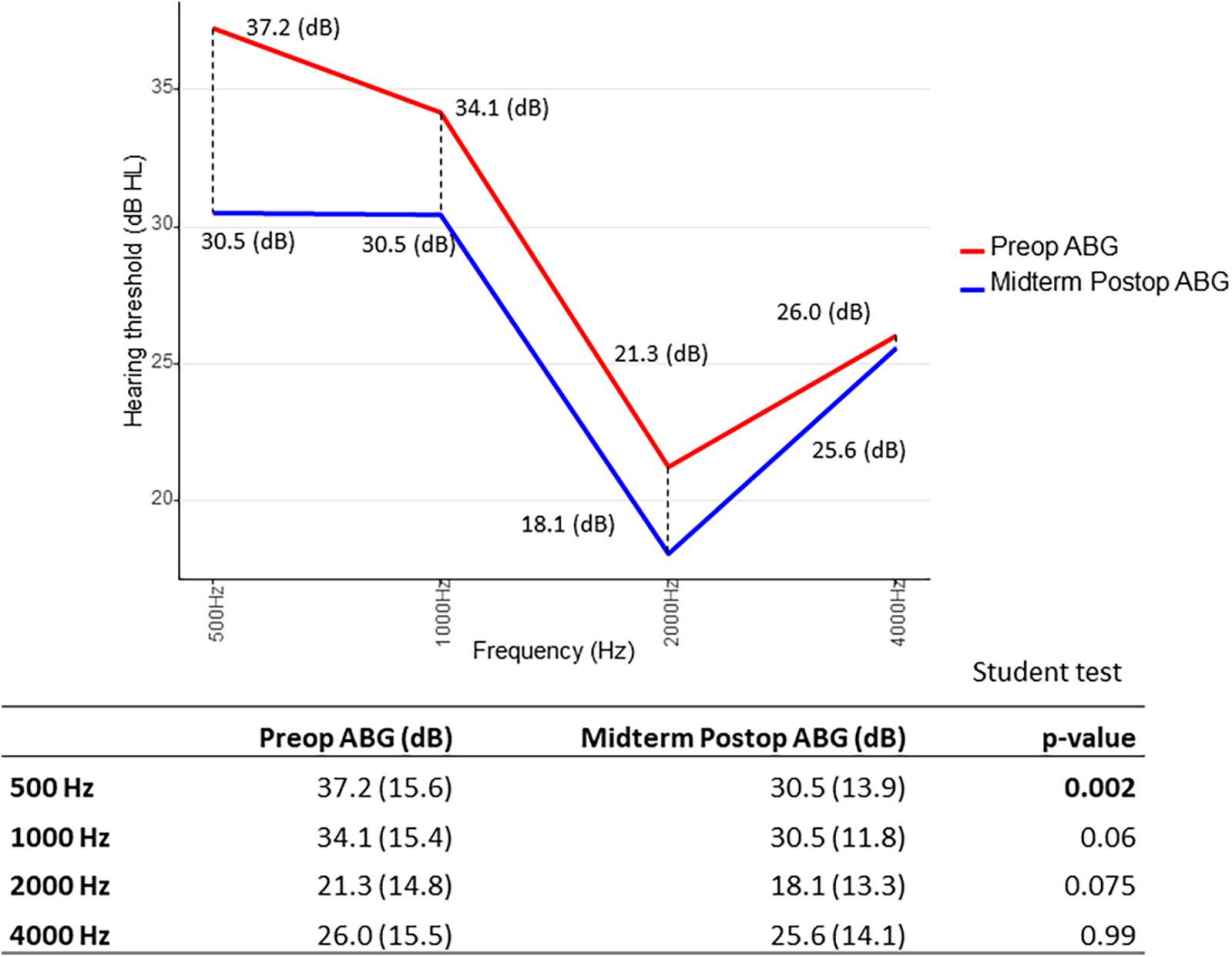
Midterm gain in air–bone gap (ΔABG) depending on frequency distribution. The *p*-value was calculated by comparing preoperative (red) and midterm postoperative (blue) air–bone gap (ABG) values at each frequency for each patient. We then determined whether the mean difference significantly differed from zero. Paired Student’s *t*-test.

Furthermore, we searched for results of ΔABG as a function of frequency distribution: for early ΔABG, gain was best at 500 and 4,000 Hz. In other terms, the difference between preoperative ABG and early postoperative ABG was statistically significant (*p* < 0.05) at 500 and 4,000 Hz, using the paired Student’s *t*-test (Figure 4). As for the midterm ΔABG, gain was best at 500 Hz (*p* < 0.05) and to a lesser extent at 1,000 Hz (*p* = 0.06) (Figure 5).

Of note, since the midterm hearing results (ΔBC, ΔAC, and ΔABG) were not statistically different from the early results (*p* > 0.05, Student’s *t*-test, for the three parameters), we studied predictive factors of hearing outcome for only the *midterm* results (8 months postoperatively) because they are clinically more relevant.

### Factors influencing auditory results

*Preoperatively* (tables not shown), we searched for predictive factors possibly influencing the initial auditory results. We divided age into three groups: 0–18 (younger), 18–60 (adult), and >60 years old (elderly). Preoperative AC and BC were better in younger patients than adult patients who in turn were better than the elderly (*p* = 0.027 for AC and *p* < 0.001 for BC, Kruskal–Wallis test). In addition, patients for whom the stapes superstructure was present had a better (smaller) preoperative ABG (*p* = 0.001).

*Postoperatively*, and regarding the midterm postoperative gain in ABG or midterm “ΔABG,” we found one factor positively influencing this parameter, namely, absence of posterior tympanotomy (*p* = 0.046) (Table 3).

**Table 3:** Analysis of factors possibly influencing midterm gain in air–bone gap (midterm ΔABG).

Parameter analyzed	Modality	Median in dB (Q1–Q3)	*p*-value midterm ΔABG
Age (years)	0–18	5 (−3.8 to 15)	0.331
	18–60	0 (−5 to 12.8)	
	60–100	0 (−5.6 to 3.1)	
Ear	Right	4.4 (−2.2 to 13.8)	0.147
	Left	0 (−5.9 to 8.8)	
Surgery	Primary	2.5 (−3.1 to 13.1)	0.454
	Revision	0 (−5 to 11.2)	
Technique	CWU	1.2 (−6.2 to 11.6)	0.221
	No mastoidectomy	3.1 (0 to 17.8)	
Posterior tympanotomy (within CWU mastoidectomy)	No	1.9 (−4.1 to 14.1)	**0.046**
	Yes	−2.5 (−7.5 to 5.6)	
Type of ossiculoplasty	Stapes augmentation	1.2 (−4.4 to 12.5)	0.287
	OtoMimix	0 (−11.9 to 0)	
	PORP	−2.5 (−6.9 to 5.6)	
	TORP	6.2 (−1.2 to 12.5)	
	Incus transposition	14.4 (−1.2 to 29.7)	
Type of ossiculoplasty without small subgroups	Stapes augmentation	1.2 (−4.4 to 12.5)	0.292
	PORP	−2.5 (−6.9 to 5.6)	
	TORP	6.2 (−1.2 to 12.5)	
Stapes augmentation	No	2.5 (−5 to 12.5)	0.939
	Yes	1.2 (−4.4 to 12.5)	
Middle ear mucosa	Inflammatory	3.8 (−1.2 to 15)	0.283
	Normal	0 (−6.2 to 10)	
Malleus handle	Absent	0 (−5 to 2.5)	0.261
	Present	3.1 (−4.7 to 13.4)	
Stapes superstructure	Absent	6.2 (−1.2 to 12.5)	0.211
	Present	0 (−6.2 to 11.2)	
Mastoid obliteration	No	5 (0 to 15)	0.101
	Yes	0 (−6.2 to 10.6)	
	Tragus		
Preoperative complications	No	3.8 (−2.5 to 13.8)	0.078
	Yes	−1.2 (−7.5 to 5)	
Postoperative complications	No	2.5 (−5 to 12.5)	0.971
	Yes	0 (−2.5 to 11.2)	

One parameter was found to positively influence hearing results: absence of posterior tympanotomy (*p* = 0.046). Kruskal–Wallis test.

Bold values are statistically significant (*p* < 0.05).

**Figure 6:**
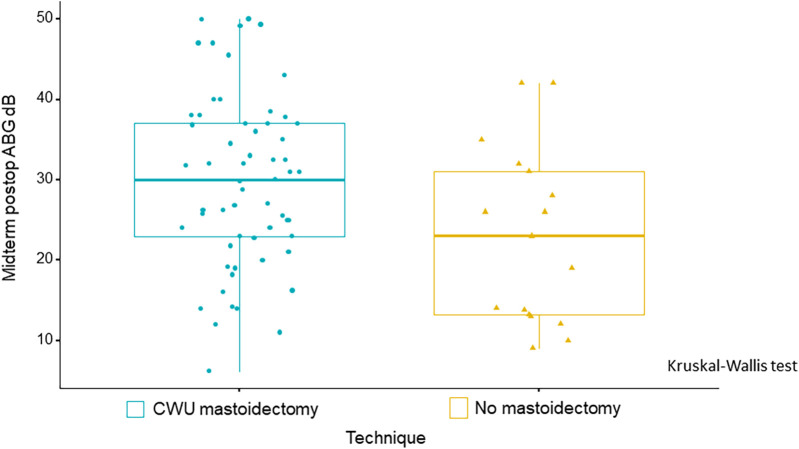
Boxplots illustrate a statistically significant improvement in midterm postoperative ABG, with smaller ABG values observed in patients who did not undergo mastoidectomy as compared with those who underwent CWU mastoidectomy. *p* = 0.014, Kruskal–Wallis test.

If we consider the midterm postoperative ABG alone (absolute value without the Δ), one factor was found to have an impact on this parameter, namely, the surgical technique: absence of mastoidectomy yielded better auditory results than mastoidectomy (i.e., CWU mastoidectomy) (*p* = 0.014) (Table 4, Figure 6).

**Table 4:** Analysis of factors possibly influencing midterm postoperative ABG alone.

Parameter analyzed	Modality	Median dB (Q1–Q3)	*P*-value midterm postop ABG
Age (years)	0–18	30 (22.5–36.2)	0.088
	18–60	23.8 (17.2–28.1)	
	60–100	27.5 (20.6–35.6)	
Ear	Right	25.6 (20.3–35)	0.691
	Left	26.2 (16.6–32.5)	
Surgery	Primary	25 (16.2–31.9)	0.127
	Revision	27.5 (22.5–35)	
Technique	CWU	26.2 (21.2–35)	**0.014**
	No mastoidectomy	17.5 (12.5–26.6)	
Posterior tympanotomy (within CWU mastoidectomy)	No	25.6 (18.1–32.8)	0.064
	Yes	30 (25.3–36.9)	
Type of ossiculoplasty	Stapes augmentation	26.2 (16.9–34.4)	0.328
	OtoMimix	27.5 (16.2–28.1)	
	PORP	22.5 (15.6–26.2)	
	TORP	30 (22.5–35.6)	
	Incus transposition	30 (24.1–34.1)	
Type of ossiculoplasty without small subgroups	Stapes augmentation	26.2 (16.9–34.4)	0.138
	PORP	22.5 (15.6–26.2)	
	TORP	30 (22.5–35.6)	
Stapes augmentation	No	26.2 (20–32.5)	0.909
	Yes	26.2 (16.9–34.4)	
Middle ear mucosa	Inflammatory	26.2 (17.5–32.5)	0.965
	Normal	26.2 (19.4–34.4)	
Malleus handle	Absent	26.9 (23.4–28.4)	0.866
	Present	26.2 (17.5–33.8)	
Stapes superstructure	Absent	30 (22.5–35.6)	0.121
	Present	25 (17.5–32.5)	
Mastoid obliteration	No	22.5 (15–33.8)	0.146
	Yes	26.2 (22.5–32.5)	
Preoperative complications	No	25 (16.2–32.5)	0.115
	Yes	30 (22.5–35)	
Postoperative complications	No	26.2 (20–32.5)	0.773
	Yes	35 (11.2–38.1)	

One parameter was found to positively influence hearing results (smaller ABG) in a statistically significant manner: no mastoidectomy (*p* = 0.014). Kruskal–Wallis test.

Bold values are statistically significant (*p* < 0.05).

Although of less clinical relevance, especially since BC level changes were minimal postoperatively, we sought factors that could influence the midterm postoperative gain in BC or midterm ΔBC and found no factor influencing this parameter (table not shown).

Regarding the midterm postoperative gain in AC or midterm “ΔAC,” we found only one factor significantly influencing this parameter, namely, primary surgery as opposed to revision surgery (*p* = 0.014) (Table 5).

**Table 5:** Analysis of factors possibly influencing midterm gain in air conduction (midterm ΔAC).

Parameter analyzed	Modality	Median dB (Q1–Q3)	*p*-value midterm ΔAC
Age (years)	0–18	−13.4 (−16.4 to 4)	0.134
	18–60	−3 (−7.4 to 5)	
	60–100	2 (−2.1 to 8.5)	
Ear	Right	−4 (−14.7 to 2.9)	0.21
	Left	−1.6 (−7.4 to 9.6)	
Surgery	Primary	−6.1 (−15.2 to 5)	**0.014**
	Revision	1.6 (−1.4 to 5)	
Technique	CWU	−1.2 (−12.7 to 6)	0.248
	No mastoidectomy	−6 (−10 to 0.2)	
Posterior tympanotomy (within CWU mastoidectomy)	No	−5 (−14.5 to 5.6)	0.286
	Yes	1 (−3 to 8.5)	
Type of ossiculoplasty	Stapes augmentation	−5 (−12.8 to 1.6)	0.074
	OtoMimix	10 (3.5 to 25.5)	
	PORP	1 (−4.5 to 5.6)	
	TORP	−7 (−14 to 8.5)	
	Incus transposition	−14.1 (−22.2 to −8.8)	
Type of ossiculoplasty without small subgroups	Stapes augmentation	−5 (−12.8 to 1.6)	0.142
	PORP	1 (−4.5 to 5.6)	
	TORP	−7 (−14 to 8.5)	
Stapes augmentation	No	−1 (−11 to 7.4)	0.236
	Yes	−5 (−12.8 to 1.6)	
Middle ear mucosa	Inflammatory	−5 (−9 to 5)	0.733
	Normal	−1.2 (−13 to 5)	
Malleus handle	Absent	−0.5 (−11.6 to 4.1)	0.937
	Present	−3 (−12.1 to 5.3)	
Stapes superstructure	Absent	−7 (−14 to 8.5)	0.644
	Present	−1.2 (−8.8 to 5)	
Mastoid obliteration	No	−4 (−10 to 5)	0.437
	Yes	−1.2 (−11.8 to 6.3)	
Preoperative complications	No	−3 (−13 to 5)	0.697
	Yes	−1.2 (−8.8 to 4)	
Postoperative complications	No	−2 (−12.4 to 5)	0.691
	Yes	−6 (−9.5 to 10)	

One parameter was found to significantly influence hearing results: primary surgery as opposed to revision surgery (*p* = 0.014). Kruskal–Wallis test.

Bold values are statistically significant (*p* < 0.05).

Now, if we consider the midterm postoperative AC alone, three factors were found to positively influence the results: age ≤ 60 vs. age > 60 (*p* = 0.043), primary surgery vs. revision surgery (*p* = 0.006), and absence of mastoidectomy (*p* = 0.015) (Table 6).

**Table 6:** Analysis of factors possibly influencing midterm air conduction (midterm AC).

Parameter analyzed	Modality	Median dB (Q1–Q3)	*p*-value midterm postop AC
Age (years)	0–18	41 (30–51.4)	**0.043**
	18–60	39 (30.6–49.9)	
	60–100	57.5 (41.3–67.5)	
Ear	Right	46.1 (31.6–55.5)	0.569
	Left	41 (31.8–52.6)	
Surgery	Primary	36 (30–51)	**0.006**
	Revision	46.6 (41.9–59.5)	
Technique	CWU	44.4 (34.5–58.5)	**0.015**
	No mastoidectomy	35 (26.2–45.2)	
Posterior tympanotomy (within CWU mastoidectomy)	No	42 (31.1–53.5)	0.078
	Yes	51 (38.8–59)	
Type of ossiculoplasty	Stapes augmentation	40 (31.2–57.6)	0.792
	OtoMimix	40 (36–45.5)	
	PORP	37 (29–52.5)	
	TORP	46.2 (38.8–53)	
	Incus transposition	45.5 (39–46.7)	
Type of ossiculoplasty	Stapes augmentation	26.2 (16.9–34.4)	0.138
	PORP	22.5 (15.6–26.2)	
	TORP	30 (22.5–35.6)	
Stapes augmentation	No	43.8 (32.9–51.8)	0.978
	Yes	40 (31.2–57.6)	
Middle ear mucosa	Inflammatory	38 (31.2–46)	0.322
	Normal	45 (33–56.2)	
Malleus handle	Absent	44.4 (38.8–54.1)	0.77
	Present	42 (31.2–53.2)	
Stapes superstructure	Absent	46.2 (38.8–53)	0.262
	Present	40 (31–54)	
Mastoid obliteration	No	38.9 (29.5–51.4)	0.315
	Yes	43.8 (34–56)	
Preoperative complications	No	38.8 (30–51)	0.12
	Yes	45 (42–57.5)	
Postoperative complications	No	42 (31.6–54.5)	0.671
	Yes	47 (36–51.4)	

Three factors were found to positively influence the results: younger age (*p* = 0.043), primary surgery as opposed to revision surgery (*p* = 0.006), and absence of mastoidectomy (*p* = 0.015). Kruskal–Wallis test.

Bold values are statistically significant (*p* < 0.05).

Finally, regarding the type of ossiculoplasty, and since the number of patients undergoing incus transposition and OtoMimix bridging is very small (four and three patients, respectively), we repeated the analyses of the abovementioned parameters (midterm ΔABG, midterm postoperative ABG, midterm ΔAC, and midterm postoperative AC) without these two subgroups and still found no influence of the type of ossiculoplasty on the postoperative hearing outcomes (*p* > 0.05 in all cases) (Tables 3–6).

## Discussion

The primary goal of surgery for cholesteatoma is disease eradication, with hearing restoration as a secondary objective. The analyzed cases in this study involved patients who initially presented with an aggressive cholesteatoma with 28 complications before surgery. In fact, the present authors practice in an academic, tertiary referral center, and the referred patients often present with complex and challenging cases. This may explain the modest hearing gains observed in our study, especially considering that over one-third of our patients had undergone prior surgery, mostly in other institutions. This being said, our auditory results are comparable to those reported by other highly experienced surgical teams who reported on results of ossiculoplasties in cholesteatoma surgery (3, 4, 6), although they are slightly inferior to those reported in others (7–9).

Although not the primary focus of our study, it is worth mentioning that the overall recidivism rate in our series (16/88  =  18.2% at 1–3 years, all residual cholesteatomas) falls within the range reported in other studies—including both pediatric and adult patients—which varies from 1.4% to 23.4% over follow-ups of 13 to 36 months (5, 7, 9, 10).

If we consider only the adult population in our study, the residual rate was 14.7%; this is also comparable with the rate of recidivism (residual and recurrent cholesteatoma) reported in a study of adult patients only, which ranged from 12.7% to 21.3% over nearly 3 years of follow-up (10).

Now if we consider only the pediatric population in our study, the residual rate almost doubled (30%), which is a little less elevated than the rates of a study analyzing only pediatric patients, but with a much longer follow-up period (39% residual disease at 3 years and 45% at 6 years) (11).

It is also important to mention that the extrusion rate (1.1%) of titanium prostheses in our work was as low as other published series (0%–3.5%) using, among others, titanium prostheses, with longer follow-up periods (1–5 years) (6, 7, 9, 12–14).

Most studies focusing on auditory results after ossiculoplasty in cholesteatoma surgery do not mention the rate of surgical complications. Our rate is relatively low, given the high rate of preoperative complications (15).

We sought prognostic factors for hearing outcomes. The presence of the stapes superstructure had a positive influence on preoperative ABG. One interpretation is that when the stapes is still present preoperatively, the disease is less erosive (less osteolytic). Regarding the midterm postoperative gain in ABG or midterm “ΔABG,” the only factor positively influencing this parameter (bigger Δ) was the absence of posterior tympanotomy. This could be hypothetically explained by the fact that cases necessitating a posterior tympanotomy reflect more extensive disease in the mesotympanum and retrotympanum (sinus tympani). When considering the midterm ABG alone, the absence of mastoidectomy was the only predictive factor of good hearing results. This positive effect is probably related to less extensive disease, particularly in the epitympanum and antrum, which obviates the need for mastoidectomy. This was also found by other authors (16).

Considering the midterm “ΔAC,” the only predictive factor for a good hearing result (bigger Δ) was the primary surgery vs. revision surgery. This positive effect could be explained by the fact that revision surgery reflects recurrent and aggressive disease and yields more fibrosis and tympanosclerosis than primary surgery. This favorable effect of primary surgery was observed in other studies (7, 13, 16). When considering the midterm postoperative AC alone, there were three favorable predictive factors (for a smaller postoperative AC), namely, younger age, primary surgery, and absence of mastoidectomy. The positive effect of the younger age could be interpreted by the fact that younger patients already had better hearing (smaller AC) preoperatively.

It is worth noting that in our study, the presence or absence of the stapes superstructure had no significant effect on *postoperative* hearing results, similar to other studies (16, 17) and contrary to others (13, 18). Moreover, the presence or absence of the malleus handle did not have any impact on hearing results in our data, although the necessity of its removal was generally associated with more aggressive disease. This is also shared by some authors (6, 9) but contradicted by others (12, 14, 16, 19).

In the literature, predictive factors having an impact on hearing outcome after cholesteatoma surgery with ossiculoplasty are highly variable (3, 6–9, 20). Fukuda et al. found that in attic cholesteatoma, postoperative hearing outcome worsened with an increase in staging of the disease (I to II to III), according to the European Academy of Otology and Neuro-Otology/Japanese Otological Society (EAONO/JOS) classification and with the involvement of the stapes with cholesteatoma or granulation tissue (20). Using costal cartilage homografts, Quaranta et al. (8) noticed no difference between PORP and TORP groups in terms of postoperative ABG levels. Contrarily, using autograft and hydroxyapatite ossicular prostheses, Sevik Elicora et al. (3) found that PORPs yielded significantly lower postoperative ABG levels than TORPs did, but no difference was found with regard to hearing gain, probably due to the relatively better preoperative hearing levels in patients with an intact stapes.

Espitalier et al. (7) analyzed patients undergoing cholesteatoma surgery with an intact stapes and observed that the success rate (ABG ≤ 20 dB) was significantly higher in primary surgery than in revision surgery. Acke et al. (6) analyzed hearing results of ossiculoplasties in primary cholesteatoma surgery with an intact stapes; they found that incus transposition and CWU mastoidectomy resulted in a lower residual ABG after surgery. Querat et al. (9) analyzed hearing data of ossiculoplasties in CWU tympanoplasties for cholesteatoma with an intact stapes. They found no statistical difference in postoperative hearing results between the cartilage group (stapes augmentation) and the hydroxyapatite PORP group.

Some studies analyzed hearing results after ossiculoplasty and included other pathologies in addition to cholesteatoma, the latter being the most frequently encountered pathology, though. De Vos et al. (12) and Truy et al. (14) observed that the presence/absence of the malleus handle and the status of the middle ear mucosa were the only parameters that had a statistically predictive value on postoperative hearing. Furthermore, using the Austin–Kartush classification of ossicular status, De Vos et al. (12) noted that the best hearing results were obtained in Class B (malleus present M+, stapes absent S−), while Austin et al. (18) found a positive role if the stapes was present, in addition to the malleus. Schmerber et al. (13) noticed that the good prognostic factors for postoperative hearing restoration were the presence of the stapes superstructure, preservation of the canal wall during mastoidectomy (CWU), primary surgery, and the presence of the malleus handle. Gelfand and Chang (21) noticed that the main bad prognostic factor in all ossiculoplasties using titanium prostheses was cholesteatoma (PORP and TORP), as opposed to other middle ear pathologies; this was not found in hydroxyapatite prostheses. Dornhoffer and Gardner (16), in their analysis of ossiculoplasty in different pathologies, found that the absence of ear drainage, normal mucosa, presence of the malleus, absence of mastoidectomy, and primary surgery were statistically significant predictors of good hearing outcome. In a nice systematic review of the literature, Blom et al. (17) noticed that the only parameter predictive of hearing outcome in surgery for chronic otitis media with or without cholesteatoma was the malleus status: positive predictor if present, regardless of the stapes condition. Of note, they found no study analyzing the presence or absence of the incus.

Our work has many limitations. First, this is a retrospective study, as were the aforementioned studies. However, conducting a prospective—and particularly a randomized—study in patients with cholesteatoma is extremely challenging due to the wide variability in ossicular damage, disease extension, and staging. Second, when the stapes was present, different types of ossicular reconstruction were used, in contrast to other studies where a single type of prosthesis was consistently employed (7, 8, 13, 16, 20). We used chiefly stapes–cartilage assembly and PORP. This adds further heterogeneity in interpreting the results and stems from the retrospective design of the study and, more importantly, from the individualized approach to ossiculoplasty. Third, 76 out of 88 cases were analyzed at 6–9 months (median 8) postoperatively because 12 cases lacked audiometry at 8 months of follow-up, due to patients living far from our institution, being followed by another otologist, or returning only 12–18 months after surgery (for postoperative MRI). This is also due to the retrospective nature of the present study and thus the absence of a pre-established follow-up schedule. Fourth, we did not perform multivariate analyses but only univariate ones, because the main goal of our analyses was descriptive. In addition, the small number of significant variables (parameters) in univariate analysis as well as the small number of patients per variable and per modality was not in favor of multivariate analysis. Fifth and last, many variables (ΔABG, ABG, ΔBC, ΔAC, AC) were tested because these are the parameters that the otologists measure pre- and postoperatively. The high number of variables in a relatively small sample size can increase the risk of Type I error (risk of false positives) which is also a limitation of our study.

## Conclusion

The auditory results after surgery for cholesteatoma were fair in our hands, at least in part because of the initial preoperative aggressiveness and extension of the disease. The rates of residual cholesteatoma and prosthesis extrusion were comparable to other studies published in the literature, and the rate of surgical complications was relatively low.

To summarize, a younger age and the presence of stapes superstructure were predictive factors of better preoperative hearing. Postoperatively, a younger age, the absence of mastoidectomy, the absence of posterior tympanotomy, and primary surgery were generally predictors of good hearing outcome. We found no effect from other factors, particularly the presence or absence of the malleus, which is often emphasized in other articles. Indeed, results in the literature are very diverse and sometimes contradictory. This stems from the highly variable degrees of disease extension and osteolysis, the surgical techniques including the ossiculoplasty, and the materials of ossiculoplasty used across studies.

## Data Availability

The raw data supporting the conclusions of this article will be made available by the authors, without undue reservation.
